# High-precision object detection network for automate pear picking

**DOI:** 10.1038/s41598-024-65750-6

**Published:** 2024-06-28

**Authors:** Peirui Zhao, Wenhua Zhou, Li Na

**Affiliations:** 1 College of Food Science and Engineering, Central South University of Forestry and Technology, Changsha, 410004 China; 2 Changsha Vocational and Technical College of Commerce and Tourism, Changsha, 410004 China

**Keywords:** Agricultural intelligence, Object detection, Deep learning, YOLOv8, Non-maximum suppression, Environmental sciences, Planetary science

## Abstract

To address the urgent need for agricultural intelligence in the face of increasing agricultural output and a shortage of personnel, this paper proposes a high precision object detection network for automated pear picking tasks. The current object detection method using deep learning does not fully consider the redundant background information of the pear detection scene and the mutual occlusion characteristics of multiple pears, so that the detection accuracy is low and cannot meet the needs of complex automated pear picking detection tasks. The proposed, High-level deformation-perception Network with multi-object search NMS(HDMNet), is based on YOLOv8 and utilizes a high-level Semantic focused attention mechanism module to eliminate irrelevant background information and a deformation-perception feature pyramid network to improve accuracy of long-distance and small scale fruit. A multi-object search non-maximum suppression is also proposed to choose the anchor frame in a combined search method suitable for multiple pears. The experimental results show that the HDMNet parameter amount is as low as 12.9 M, the GFLOPs is 41.1, the mAP is 75.7%, the mAP50 reaches 93.6%, the mAP75 reaches 70.2%, and the FPS reaches 73.0. Compared with other SOTA object detection methods, it has the transcend of real-time detection, low parameter amount, low calculation amount, high precision, and accurate positioning.

## Introduction

The pear is one of the five largest fruits in the world. Due to its high economic value, high nutritional and health value, and multiple uses for fresh food and processing, it is very popular among producers and consumers^1^. Furthermore, because of its strong adaptability, early fruiting, high yield and long economic life, pears are cultivated in a wide range of countries throughout the world, and have played an important role in promoting rural economic development and increasing farmers’ income over many years^2^. In the pear production line, pear picking is an indispensable part, and fruit picking is a labor-intensive exercise^3^. At present, pear picking operations in many parts of the world are still done manually, with low automation, high labor-intensive intensity, low picking efficiency and high cost^4^. At the same time, due to the development of modern industries and social economy in various countries, more and more laborers are moving from rural areas to cities, resulting in a shortage of agricultural productivity. The supply of productivity is in short supply, and the contradiction between the increase of its output and the shortage of labor force has become increasingly prominent^5^. Therefore, it is urgent to introduce automation technology into fruit picking, and the development of fruit picking robots can improve agricultural production efficiency and competitiveness of agricultural products, etc^6^. In the research and development process of the picking robot, the vision system is its core component, and is also an important and difficult point of research and development. It is responsible for detecting and locating the fruit, fruit stem, and fruit calyx, and provides the obtained position information to the central processing unit to guide the robot. Planning and completing picking operations^7^. Due to the complex growing environment of pears, usually in the wild or in plantations, there are factors such as overlapping occlusion, light changes, and different shapes and colors of different pear varieties, which bring challenges to conventional object detection models. For the development of fruit picking robots caused certain restrictions^8^. At present, some progress has been made in the identification and detection tasks of fruits, but the high-precision identification and positioning of pears in the complex environment of the orchard can enhance the adaptability and robustness of the visual system in complex environments, and improve the ability of the neural networks. Accuracy will help the popularization and application of pear picking robots, resolve the contradiction between labor shortage and increasing fruit production year by year, reduce fruit production costs, and improve fruit picking efficiency.

The recognition of the visual system of traditional robot picking is based on sensors. This picking system has good efficiency for fruit picking in simple scenes, but the anti-interference ability of the sensor is extremely poor. When the external environment such as weather changes, the recognition of sensor ability of the pear may drop sharply, and the sensor can only pick specific fruits, and it is not migratory. However, there are many varieties of fruit pears, and their basic attributes such as size and shape are still quite different. Studying specific picking systems in different environments will consume huge research costs and production costs, resulting in a lot of waste of resources^9^. For the above reasons, traditional sensor-based fruit identification systems have not been popularized and have little effect in agricultural production. With the development of large datasets and large model architectures, deep learning has opened up ideas for solving such problems. The robot system that combines deep learning algorithms with robotic arms to achieve target positioning has been verified in many other fields. In terms of target recognition, methods such as K-means clustering^10^, color difference method^11^ and fuzzy C-means method^12^ commonly used in machine learning require researchers to manually select appropriate features to achieve specific effects. Compared with the sensor method, the feature selection has a significant improvement, but due to the single feature and fixed target, these methods have poor mobility and weak generalization ability. Compared with the above methods, the network can learn a log of training data from the neural network, learn suitable features from the picture, and can achieve good results for different goals. It has good generalization ability and can adapt to different task requirements. and detection of pears in different environments^13^. The deep learning method aims to learn the optimal parameters of the network model through multiple iterations of a large number of training samples, which makes the model more expressive and transferable^14^. Therefore, the application of deep learning methods to accurately identify pears and provide real-time feedback of the location information of pears to the manipulator is the preferred solution to achieve the purpose of automated picking.

**Figure 1:**
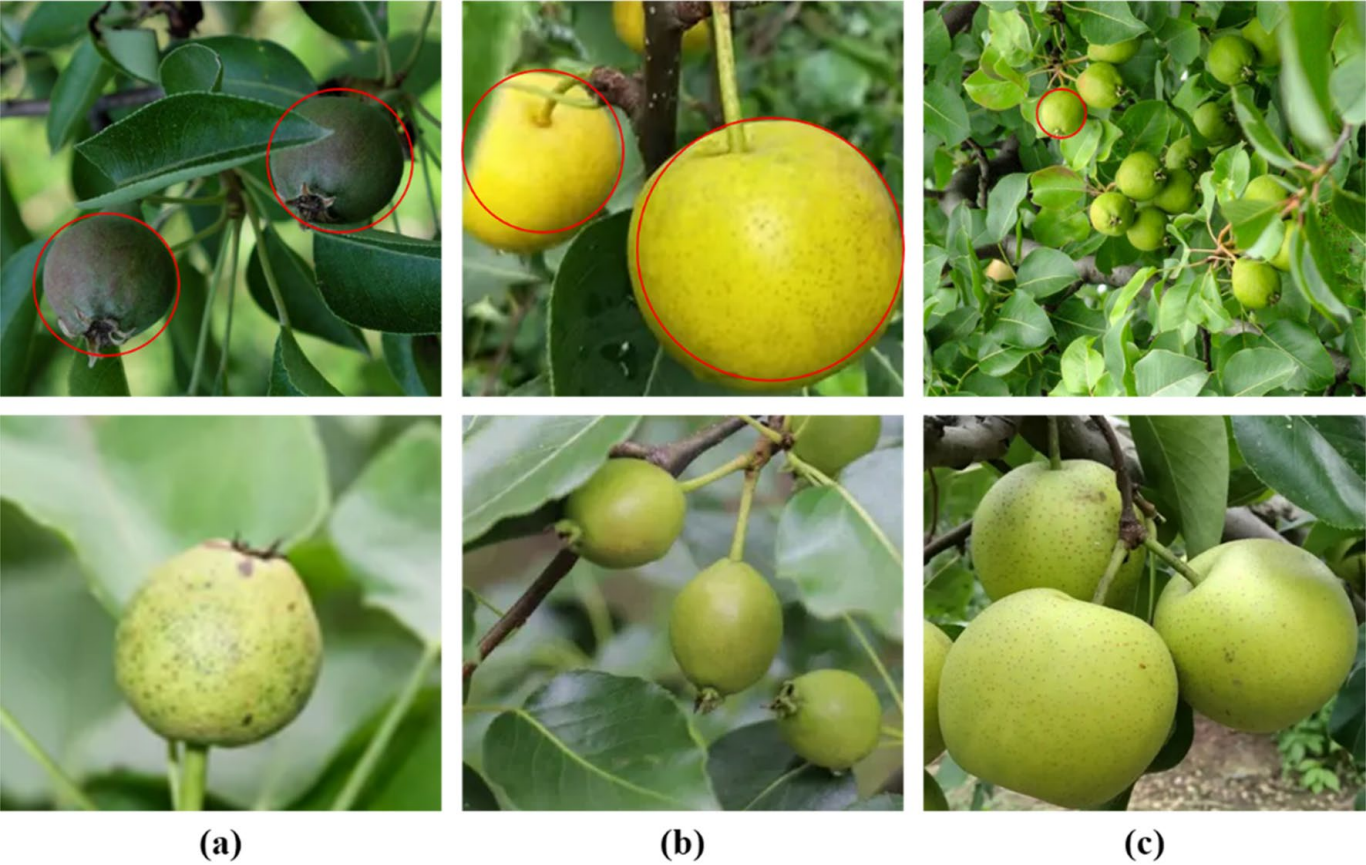
Detection challenges brought by automated picking of pears. (**a**) Irrelevant background interference and occlusion. (**b**) Size differences among different pears. (**c**) Multiple pears are difficult to locate. (The red circle indicates pear targets that are difficult to detect).

In the automated picking of fruit and pears, the accuracy of identification and positioning is the key to help the manipulator pick accurately and prevent damage to the fruit body. Therefore, there is an urgent need to design a high-precision object detection method for manipulators. Among the many object detection algorithms based on deep learning, YOLOv8 not only has a simple network structure, but also has the accuracy and real-time performance beyond the previous single-stage model in general object detection scenarios. However, in the actual automated fruit picking task of pears, YOLOv8 still has the following problems to be solved urgently(See Fig. 1). (1) The application scene of automated pear picking is complex, and there may be many occlusions of irrelevant backgrounds such as leaves and tree trunks. These objects have similar information to pears in the image, and the feature information extracted by traditional networks have weak feature extraction capabilities, so it is difficult to distinguish among them. The difference in the characteristics of the pear is likely to cause misjudgment of the position of the pear body and the calyx of the fruit stalk. (2) Due to the need for automated picking of pears, there are large differences in the scale of pears and the distance from the camera. When capturing pear information, the distance between the camera and pears is often difficult to control carefully. YOLOv8 is used for detection tasks where the distance changes too much underperformed. (3) A fruit tree often produces multiple fruit pears. When the camera captures the image of the fruit tree, the captured image usually has multiple fruit pears, and there are many cases of occlusion between the fruit pears. The Matrix NMS method of YOLOv8 detects each part of the fruit pear. The location is filtered out, and the global information and the multi-occlusion characteristics between pears are not combined, which may cause a large deviation in the object detection model used in the automated pear picking system in respect of the position predicted by the anchor frame, which means it is easy for missed or false detections to occur.

Although YOLOv8 has the above-mentioned defects in the automated picking of pears, it has achieved a breakthrough effect in the usual object detection, which guides the direction for the research on the visual system of automatic pear picking. Therefore, in order to solve the above three difficulties and improve the detection ability, this paper proposes an object detection method for automated pear fruit picking based on HDMNet. This method can be used as a visual system for automated picking and positioning, embedded in the visual. In practical application systems such as robots and automatic picking devices, our contributions are summarized as follows:

1. We propose the HDMNet model for automated pear harvesting detection based on YOLOv8 as the baseline. By introducing the advanced Semantic Focus Attention Mechanism module (HSA), we successfully eliminate irrelevant background information. Additionally, by utilizing the Deformation-aware Feature Pyramid Network (DP-FPN), we significantly enhance the accuracy of detecting distant and small target pears.
2. Addressing the issue of the lack of a recognized dataset for automated pear harvesting detection, we have collected and annotated 8363 images of pear trees in orchards. This dataset has been meticulously curated, providing an ample supply of samples for model training. Not only does this dataset facilitate the training of deep learning models in our study, but it also serves as a valuable resource for future research in this field.
3. Experimental results demonstrate that our proposed HDMNet exhibits outstanding precision and generalization performance in the task of automated pear harvesting detection. Furthermore, it boasts remarkable real-time capabilities. This finding not only validates the effectiveness of the model but also underscores the excellent trade-off between speed and accuracy, making it a viable choice for practical applications.
4. Most importantly, we have successfully integrated HDMNet into a self-constructed Internet of Things (IoT) system. This integration enables the model to operate seamlessly in actual pear harvesting tasks and contributes to the system’s refinement. This application showcases the core objective of this paper, which is to translate research findings into practical applications. It provides crucial technical support for automation and intelligence in the field of pear harvesting, offering efficient and reliable solutions that improve the efficiency and quality of pear harvesting for farmers.

## Relate work

The correct identification of the pear is the basis for the positioning of the pear in the automated picking system, and positioning can only be performed after the pear has been accurately identified. The following are the main methods used in the research on the identification and location of fruit picking: (1) Methods of manual picking and sensor identification. (2) Image processing methods: conventional deep learning methods and traditional image processing algorithms. (3) Convolutional neural network-based object detection method.

The applicability, reaction time, and cost of sensor detection algorithms are all constrained. Traditional image processing algorithms have poor feature learning capabilities, high rates of false and missing detection, or both. With the advantages of high precision, powerful feature learning capabilities, and the capacity to interpret Semantic information in complicated settings, deep learning overcomes the limitations of sensors and conventional image processing methods. Convolutional Neural Networks (CNNs) are deep learning models that are commonly used in image recognition and computer vision. Convolutional layers, pooling layers, and fully connected layers make up the basic structure of CNNs. Sliding windows are used by the convolutional layer to extract picture features. DoSWSampling is used by the pooling layer to lower the dimensionality of the data. CNN has the advantage of being able to identify local features in images and combine them into higher-level features, allowing for more accurate image recognition. When combined with robot control methods, the object detection methods based can be used as the robot’s vision system to help the robot use the mechanical arm to grasp accurately, resulting in an efficient and high-precision picking process of which the accuracy of object detection greatly affects the final effect of automated picking. Therefore, this paper aims to study a deep learning-based object detection algorithm for automated picking of robotic pears.

The two simplest methods are manual detection and sensor detection. The manual detection method relies on agricultural personnel to manually pick and judge whether the fruit is ripe according to their own experience, which is highly dependent on the experience of the personnel, and is time-consuming and labor-intensive. Sensor method detection mainly includes force sensor and laser sensor, people such as Shamshiri designs and develops a kind of robot apple picker, and has used various mechanical devices such as mechanical arm, gripper and sensor^15^. However, due to the sensor’s distance, shadow, and angle, the surrounding environment can influence its judgment of quality and brightness. Due to the sensor’s internal design, there will be a noticeable concurrent temporal delay. These sensors have a constrained number of uses and are also expensive and challenging to use.

In order to correctly identify fruits for automated picking, researchers have extensively studied image processing techniques for fruit identification. Early research primarily focused on analyzing the spatio-temporal sequence relationship in image sequences using traditional image processing algorithms. Shebiah effectively fused the color and texture features of the fruit ^16^, and completed the identification of different fruits through the minimum distance classification based on the statistical properties of wavelet transform, and proved the effectiveness of this method for the identification of various fruits. Xiang et al.^17^ proposed a tomato bite recognition algorithm based on circular regression, which eliminated abnormal points by calculating the curvature of the extracted closed edge points, and then used the circular regression method to identify occluded fruits. The recognition accuracy rate reached 90.9%, and the recognition effect on tomatoes with medium and heavy occlusions is poor. Bansal et al.^18^ developed a new technique to detect unripe green citrus fruits from outdoor color images by comparing the FFT leakage values of fruits and leaves to obtain thresholds. Gartica et al.^19^ used a neural network to identify olives and overlapping olives, but the simple neural network still had many false positives due to the varying interweaving of olive lines. Nanaa et al.^20^ detected the elliptical shape feature in the input mango image through random Hough transform, and used the backpropagation neural network to classify the mango. The detection rate of the fruit with clear appearance is high, but the overlapping occlusion phenomenon is significant. The effect of fruit detection has declined. Rizon et al.^21^ used random Cui Dachang arch to find the best ellipse fitting of the target fruit area in the binary image, and identified the occluded fruit by combining texture feature parameters and morphological operators. Rachmawati et al.^22^ combined multimodal features and fruit grade attributes to propose a hierarchical multi-feature classification system for identifying different kinds of fruits, but the classification boundaries are not clear and there are many false detections. The aforementioned methods have high computational complexity in terms of feature selection and high uncertainty in the automated picking process, resulting in poor adaptability to complex and changing environments.

Over the past few years, convolutional neural networks have made considerable advancements in deep learning, and their advantages in feature learning have opened up fresh views on the autonomous pear choosing difficulty. Several well-known deep neural networks have recently made significant advancements in the field of object detection. During the past few years, some traditional networks, such as VGGNet^23^, GoogleNet^24^, ResNet^25^, etc., with the assistance of the residual structure to properly solve the problem of gradient disappearance and gradient explosion, the depth of the model has rapidly changed from several. The number of layers have increased to hundreds of layers, thus expanding the possibilities of deep learning, the advancements of which have brought fast and accurate methods to the vision system for automated pear picking. With deep learning methods, the model can extract the effective features of the pears in the image, so as to locate one or more pears in varied planting environments and achieve precise positioning.

The YOLO^26–34^ object detection model has evolved over several versions. Initially focused on real-time detection speed, it gradually improved accuracy through enhanced feature extraction, multi-scale training, and better handling of small objects. Recent versions like YOLOv5^31^ prioritized a balance between speed and accuracy, becoming popular for real-world applications. YOLOv8^34^ further refined this balance, using efficient modules and improved loss functions for better performance in complex scenarios, making it the chosen foundation for this study, which uses YOLOv8-s as the baseline.

In the field of object detection, in addition to the YOLO series, MobileNet SSD v2^35^, EfficientDet^36^, and ViT-based^37^ detection methods each have their own unique characteristics, providing a wide range of choices for applications in different scenarios. MobileNet SSD v2, as an improved version of SSD, achieves a lightweight model through the MobileNet v2 backbone network, making it possible to deploy on mobile devices. At the same time, the characteristics of the SSD single-stage detector ensure real-time detection, making it very suitable for applications that require rapid response, such as real-time detection on mobile devices. However, MobileNet SSD v2 still has room for improvement in small object detection, and its accuracy may be slightly inferior to larger models. EfficientDet, on the other hand, achieves excellent performance on multiple datasets through BiFPN and compound scaling methods, especially in large object detection. Its compound scaling feature allows the model to be flexibly resized to adapt to different computational resources and accuracy requirements, making it highly scalable. However, EfficientDet is difficult to train and requires careful adjustment of multiple hyperparameters. Although it has improved in small object detection compared to SSD, there is still scope for enhancement. ViT-based detection methods, as an exploration of Transformer in the field of object detection, have shown their unique advantages. The global modeling capability of Transformer enables it to capture long-range dependencies and better understand the context information of objects. In addition, ViT models pre-trained on image classification can be transferred to object detection tasks, further improving performance, although ViT models^38–40^ are usually large, computationally intensive, and may be slightly inferior to CNN in small object detection.

In summary, MobileNet SSD v2 is suitable for applications that pursue speed and are required to be lightweight, EfficientDet is suitable for applications that pursue high accuracy and scalability, while ViT-based methods provide new ideas for the application of Transformer in the field of object detection and may perform well in certain specific tasks. With the continuous development of technology, these methods are also constantly improving and will play a greater role in the field of object detection in the future.

Recent research in pear detection and recognition has seen significant advancements, driven by the increasing demand for automation in agriculture. Deep learning techniques, particularly Convolutional Neural Networks (CNNs), have emerged as the dominant approach, demonstrating remarkable accuracy and efficiency in various pear-related tasks. Mask R-CNN, a state-of-the-art instance segmentation model, has been successfully applied to pear detection in orchards, achieving high accuracy even in cluttered environments^41,42^. Researchers have also leveraged 3D stereo cameras to capture depth information, further improving detection and localization accuracy^43^. To address the computational demands of real-time processing, lightweight models have been developed, often employing techniques like model compression and pruning^44^. The ECLPOD model, based on YOLOv7, is a notable example, achieving high accuracy with low computational cost in smart agriculture applications^45^. Beyond detection, researchers have also investigated factors influencing pear disease recognition using deep learning, analyzing the impact of image resolution, disease class similarity, and network architecture on accuracy^46^. Data augmentation techniques have been explored in order to enhance the robustness of models in recognizing various pear diseases^47^. Other studies, meanwhile, have focused on specific challenges in pear detection, such as the development of algorithms for real-time pear fruit detection and counting using YOLOv4 and Deep SORT^48^. Additionally, research has been conducted on pear and apple recognition using deep learning on mobile devices, showcasing the potential for on-site applications^49^. Thermal imaging has also been explored for the detection and classification of pear bruises, with promising results achieved using modified deep learning algorithms^50^. Overall, these studies demonstrate the significant progress made in pear detection and recognition, paving the way for more efficient and automated agricultural practices. However, challenges remain, such as the need for larger and more diverse datasets, the development of robust models for different pear varieties and growth stages, and the integration of detection systems with robotic platforms for automated harvesting. Continued research in these areas is crucial for realizing the full potential of pear detection and recognition technologies in modern agriculture.

## Method

### Dataset acquisition

**Figure 2:**
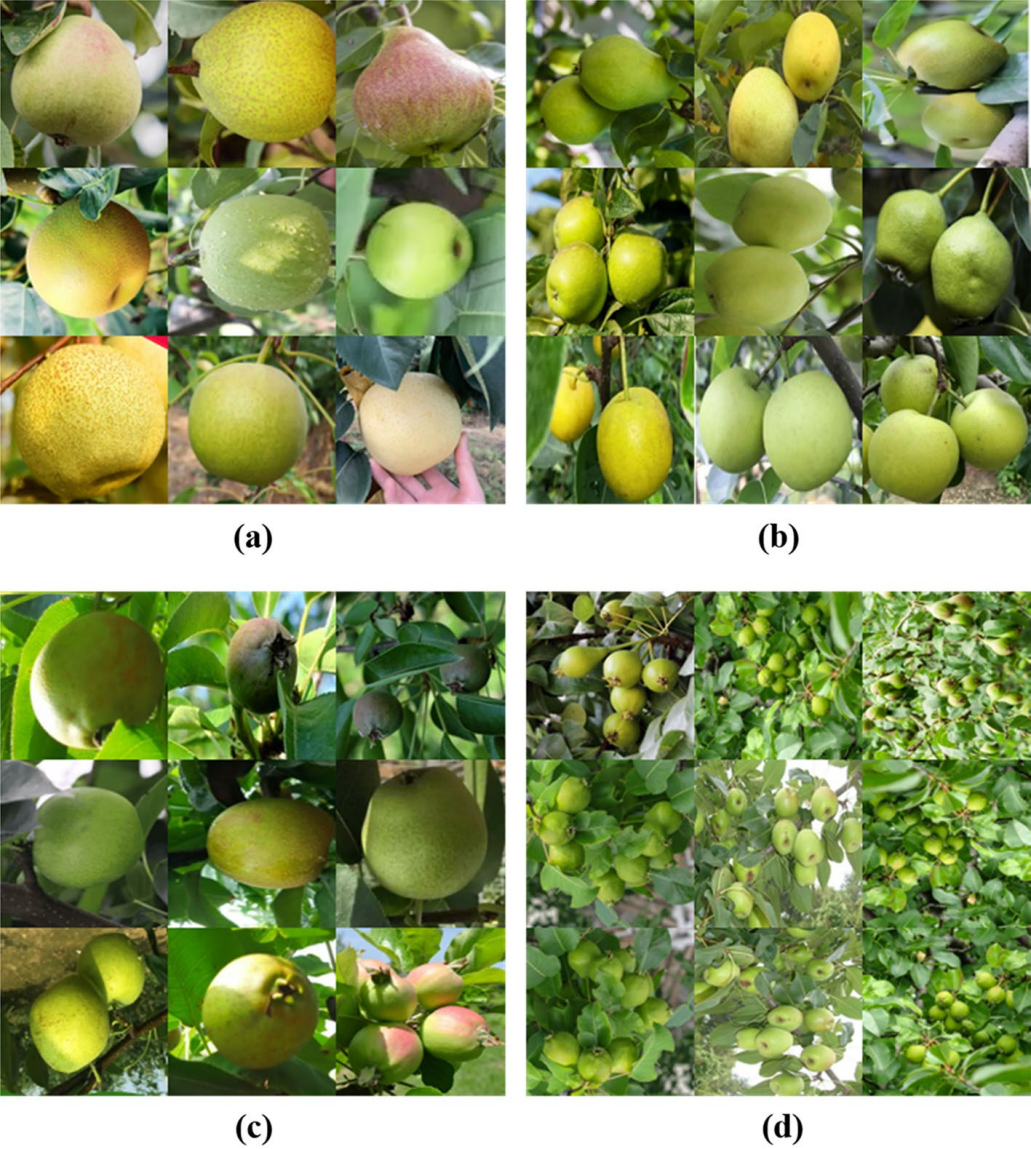
A sample of a self-made dataset; the image shows (**a**) a single pear, (**b**) several pears, (**c**) a pear with shadow interference, and (**d**) a complex image with a lot of pears and distant objects.

Due to the few images used for automated pear picking, the difficulty of labeling object detection, and the limited number of researchers in the field of object detection for agricultural automation, there is no publicly available object detection data for automated pear picking. As a result, data sets must be collected and produced prior to carrying out the research outlined in this paper. First, we use a Hikvision camera. The low-quality images obtained due to camera shake and other reasons were removed, and a total of 8363 images and 56,406 labels of fruit and pears in different postures, positions and climate environments in fruit trees and farmland were obtained (see Fig. 2 for a data set example). These pictures are images obtained in the orchard. The composition of the data set is diversified, covering the various forms of pears in the automated picking scene and the variety of the number of pears in the pictures.

**Figure 3:**
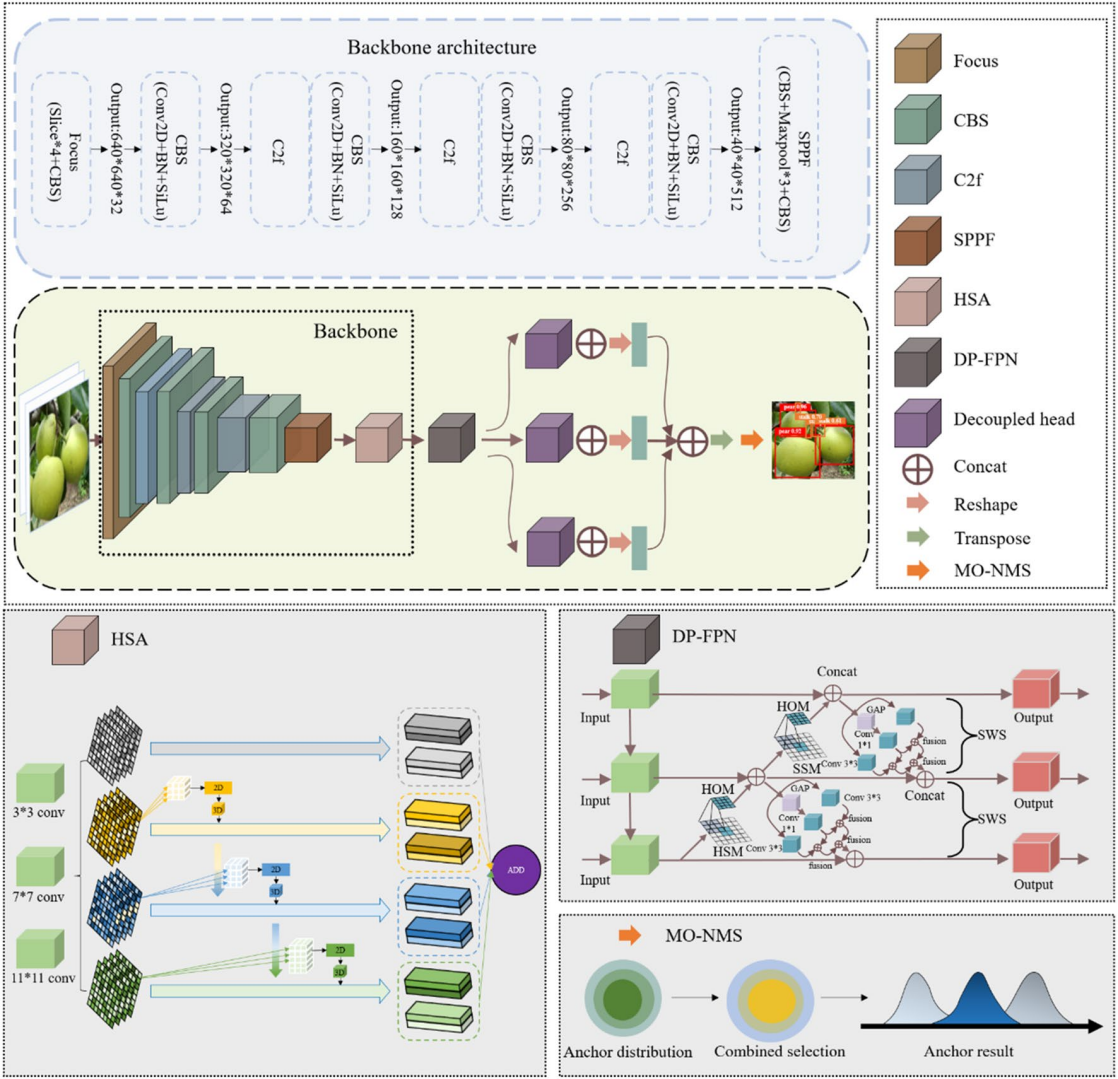
The structure of HDMNet.

To aid research, the high-definition image standard is unified to depict the actual automated picking scene through screening, cropping, and standardization. We use the labelIing software to label the object detection data, including position coordinates, length, and height information. LabelIing only labels VOC-format datasets. Following labeling, a dataset in VOC format is obtained and as a result, the dataset must also be converted to YOLO format. The steps to transform the dataset are depicted in Fig. 3.

### YOLOv8 architecture

YOLOv5 uses CSPDarknet53 as its backbone and the feature pyramid network to fuse image features, making it a object detection network classic. YOLOv8 maintains the basic structure of YOLOv5 and divides the network structure into four parts: input, backbone, neck, and prediction. Different from YOLOv5, YOLOv8 replaces the C3 module with the C2f. module to achieve further lightweight features/attributes, while YOLOv8 still uses the SPPF module used in YOLOv5 and other architectures. At the same time, before the picture is input to Backbone, Focus performs a slice operation on the picture, and takes a value for every other pixel in the picture to obtain four feature maps. At this stage, the information of the four feature maps is not lost, consequently the original picture’s W and H information is concentrated into the channel space, and the input channel is expanded four times, transforming the spliced image into multiple channels in comparison to the original RGB three-channel mode, and finally the picture obtained by Focus slice is passed through the convolution operation produceing a complete double-doSWSampled feature map. The Backbone network employs the CSP1 X structure, whereas the Neck network employs the CSP2 X structure. CSP1 X uses a residual component, and the residual connection of the residual component is conducive to the reuse of the extracted feature information, so it is suitable for Backbone. In addition, YOLOv8 uses VFL Loss as the classification loss, uses DFL Loss + CIOU Loss as the positioning loss, and uses the branch decoupling head to separate the classification branch and the positioning branch, alleviating the inherent conflict between classification and regression tasks. In addition, the Task-Aligned Assigner matching method design is used, which is especially suitable for the complex environment in the automated pear picking scene, with the advantage that its pixel-based features are not obvious. When using the previous loss function for regression, it is easy to lose feature information, which affects the detection effect. The main branch structure of YOLOv8’s feature flow accurately calculates the gradient through the new connection method, improves its generalization ability, and has achieved good results in the field of general object detection. Since the YOLO series models have good real-time and deployability, we choose YOLOv8 as the benchmark network in order to apply the time-speed and fruit characteristics concurrently. Although YOLOv8 has advantages over other traditional models in the automated pear picking task, the environmental variability of pear picking has brought us challenges. The detection rate of the model can be greatly improved, If it can be designed according to the characteristics of the pear picking task. Therefore, this paper improves based on YOLOv8 and proposes a object detection method HDMNet for pear picking tasks.

### High-level deformation-perception network with multi-object search NMS (HDMNet)

YOLO have performed admirably in the tasks of object detection. As the latest architecture of the YOLO series, YOLOv8 uses various methods to optimize accuracy, speed and parameter volume. At the same time, the C2f. structure is used to down-sample the spatial pyramid structure, which ensures the optimal solution of the model performance while saving the parameter amount, so it is suitable for the detection of pear petiole and calyx, which requires a trade-off of model parameters and detection performance. Generally, the fruit stalk is dark in color and slender in shape, and the calyx is similar to the color of pear itself in color and concave in shape. For object detection, placing these two components is challenging, and the direct use of the YOLOv8 model does not completely take into account the aforementioned features in the feature extraction process, making it challenging to produce satisfying results. If the model can be developed in accordance with the peculiarities of automated pear picking tasks, its practicability will be significantly increased. As a result, this paper proposes a method HDMNet for automated pear picking tasks based on the advancement of YOLOv8-s.

As shown in Fig. 3, the network can be divided into three parts: Fusionable convolution with structure reparameterization, High-level Semantic focused attention mechanism module, and Deformation perception Feature pyramid network. See the following sections for details.

#### High-level semantic focused attention mechanism module(HSA)

Attention mechanisms are used in computer vision to help models focus on the most important parts of an image. This mechanism allows the model to process large images more efficiently and reduce the demand on computing resources. Attention mechanisms are often implemented as neural network layers that learn weights to indicate which parts are more important for a given task. In the automated picking task of pears, the trunks on the pear trees are intricate and intertwined, and the pears are similar to the leaves in color, the degree of discrimination from the background is low, resulting in inconspicuous features on the image, hence the backbone used for feature extraction extracted The Semantic information of the feature map is weakly representative, and a low activation value is obtained, which may easily cause the neural network to recognize the feature map, which further affects the positioning and recognition of the model.

In order to increase the activation value of the feature map and obtain better Semantic information, this paper proposes a High-level Semantic focused attention mechanism module to learn the high-level Semantic features of pears, highlight the main body of pears, and filter out redundant information brought by complex backgrounds , so as to improve the feature extraction ability of the model.

1. Smaller convolution kernels are better at extracting information from smaller objects, while larger convolution kernels are typically more sensitive to large target objects. Finding the target is challenging, though, when there is a lot of foliage cover or a complicated background. We use convolution kernels with sizes of 3*3, 7*7, and 11*11 to increase recognition accuracy and add convolution kernel branches of various widths.
  1 $$Conv = \frac{1}{m}\sum\limits_{i = 1}^{m} {((x^{(i)} )^{T} u)^{2} = u^{T} (\frac{1}{m}\sum\limits_{i = 1}^{m} {(x^{(i)} )(x^{(i)} )^{T} } )} u$$
  $x^{(i)}$ represents the input feature information, and the outputs corresponding to the three convolution kernel branches of different sizes pass through the following structures respectively.
2. The feature map obtained after three convolution kernels is passed through the high-level Semantic focus component by the GAM structure.
  2 $$c_{i} = \sum\limits_{j = 1}^{n} {\frac{{\exp (e_{i,j} )}}{{\sum\nolimits_{k = 1}^{n} {\exp \left( {e_{ik} } \right)} }}} h_{j} (i = 1,2,3)$$
  As in formula (5), attention weights are first generated through the traditional attention allocation strategy, and the allocation of weights serves the representation information of the three convolution kernels. Fusion is then performed using the feature coupling mechanism to reduce the amount of calculation.
  3 $$coupling = \frac{{\exp (W_{s} (c_{k} ) + b_{s} )}}{{\sum\nolimits_{i,j,k} {\exp (W_{s} (c_{i,j} ) + b_{s} )} }}(c_{I} *c_{II} *c_{III} )$$
  Of these, $W_{s}$ and $b_{s}$ represent the weight and offset coefficients, which are the learnable parameters of the neural network, $c_{i}$,$c_{j}$ and $c_{k}$ represent the successive traversal of three different representation information to generate coupling tensors.
  4 $$result = concat(c_{I} ,c_{II} ,c_{III} ,coupling)$$
  Through formula (8), the feature tensor output by the multi-branch convolution is fully coupled. When the tensor is coupled, the neural network can pay more attention to the high-level Semantic information of pears, such as concave calyx and flat fruit stalk, thus improving the feature extraction ability of the neural network for pear targets.
3. Pears can be found in a variety of orchard scenes. Switchable Normalization (SN) is used in place of the conventional Batch Normalization (BN) layer to adapt to the complex and dynamic orchard background. SN statistics are used to calculate BN, LN, and IN three statistics, and six weight parameters are introduced to calculate the weighted mean and weighted variance, which serve as SN’s mean and variance. Using the softmax activation function, normalize:
  5 $$\mathop h\limits^{ \wedge }_{ncij} = \gamma \frac{{h_{ncij} - \sum {k \in \Omega w_{k} \mu_{k} } }}{{\sqrt {\sum {k \in \Omega w_{k}{\prime} \sigma_{k}^{2} + \varepsilon } } }} + \beta$$
  Among them, the input hidden convolutional layer of HDMNet can be expressed as a feature map with four dimensions $(N,C,H,W)$. These dimensions correspond to the number of channels (number of channels), the number of samples (minibatch size), channel height (height), and channel width (width). $h_{ncij}$ represents a pixel, $\mathop h\limits^{ \wedge }_{ncij}$ represents $h_{ncij}$ corresponding pixel normalized result. $w_{k}$, $w_{k}^{\prime}$ indicates the weight coefficient, $\mu_{k}$ means the mean value, $\sigma_{k}^{2}$ represents the variance. The model learns scaling factor $\gamma$, offset factor $\beta$ and $\varepsilon$.
  6 $$w_{k} = \frac{{e^{{\lambda_{k} }} }}{{\sum\nolimits_{{z \in \{ in,\ln ,bn\} }} {e^{{\lambda_{z} }} } }}$$
  $\lambda_{k}$ indicates the control variables corresponding to the three-dimensional statistics. All of the control variables are set to 1, and the learning is optimized during backpropagation. Then the softmax function is utilized to normalize the control parameters $\lambda_{k}$, and calculate the weight coefficient $w_{k}$.
4. Use the SiLU activation function rather than the more popular ReLU activation function or Sigmoid activation function to enhance the model’s learning convergence impact. The following formula is used to determine SiLU’s activation function:
  7 $$f(x) = x*sigmoid(x)$$
  This structure enables the neural network model to distinguish backgrounds with similar features to pears, improving the accuracy of detailed feature extraction of pears. The global texture features of pears, such as pears of similar color and size, as well as their spatial distribution, can now be given more attention by the neural network model.

#### Deformation-perception feature pyramid network(DP-FPN)

A Feature Pyramid Network, or FPN^29^, is a fully convolutional feature extractor that takes a single-scale image of any size as input and outputs proportionally sized feature maps at multiple levels, with the benefit that this procedure is not affected by the backbone convolutional architecture. YOLOv8 introduces C2f. on the basis of YOLOv5. However, this only improves the calculation rate of local parameters, and does not substantially improve the effect of feature fusion. It retains the one-way fusion pipeline and simply fuses the local parts of the fruit from top to bottom. Features, integrating the features of pear parts at different scales. However, the attention mechanism module at the end of the backbone above changes the focus of features, and this simple fusion method cannot make full use of its extracted features. In order to further release the gains brought by the attention mechanism module, we propose DP-FPN, which combines shallow visual features and deep Semantic data in channel and spatial dimensions, consisting of three parts: hierarchical spanning module(HSM), hierarchical optimization module(HOM), and self-weight sampling(SWS).

HSM is used to improve object cues at specific scales. Since attention heatmaps on different layers have different scale preferences, it allows HSM to generate scale-aware features

8 $$F_{k}^{{\text{o}}} = F_{k}^{i} \odot ({\text{W}} + A_{k} )$$

where $F_{k}^{i}$ and $F_{k}^{o}$ are the input feature maps and output scale-aware features, respectively, $A_{k}$ is the attention heatmap at layer k, ${\text{W}}$ represents a learnable parameter, and $\odot$ is the element-wise multiplication. It is important to note that residual connections are used to avoid degrading features around objects because contextual information can aid detection.

We propose a scale selection module to guide deep layers to pass suitable features to shallow layers, where suitable features do not cause inconsistencies in gradient computation because they are optimized towards the same class of. If objects in adjacent layers can be detected, the deeper layers will provide more features and will be optimized concurrently with the next layer. HOM can be written as follows:

9 $$P^{\prime}_{k - 1} = C_{k - 1} + f_{nu} (P_{k}^{\prime} ) \odot (f_{nu} (A_{k} ) \odot A_{k - 1} )$$

where the intersection of $A_{k - 1}$ and $A_{k}$ is obtained by $\odot$, $f_{nu}$ denoting the most recent upsampling operation, $P_{k}{\prime}$ is the merged texture of the kth layer, and $C_{k - 1}$ is the output of the (k − 1)th residual block.

In order to accurately obtain the contour information of the pear, HOM, in particular, serves as a scale selector. HOM introduces Scale-Invariant Feature Transform (SIFT) to the pixel change from the foreground to the background of the image caused by the image caused by the pear boundary. In the large field image of the automated pear-picking mission, complex backgrounds are usually introduced with more noise than natural scene images, which will cause greater changes in pixel gradients and bring difficulties to reverse propagation of neural networks. SIFT is a algorithm for detection, description and matching images. It is constant for uniform scaling, direction, and light changes. Even under miscellaneous and partial blocking, you can recognize objects steadily. In the HOM, the SIFT image (such as in a multi-scale input image) for each input of the convolution layer is processed. In this way, the output of the first volume of the accumulated layer is a common CNN feature diagram. After all, using SIFT images as input provides local rotation unchanged features. As a result, HOM can position each key point on the position and scale of the candidate key points, and better integrate multi -scale representation. See formulas (10) for HOM’s calculation steps.

10 $$D(x) = k(D + \frac{{\partial D^{T} }}{\partial x}x + \frac{1}{2}x^{T} \frac{{\partial^{2} D}}{{\partial x^{2} }}x) + b$$

Additionally, partial occlusions in these photos allow certain objects to be labeled with just visible sections, which causes detectors to treat parts of persons as full people, particularly when the dataset is limited. Based on these factors, we suggest SWS to improve detector generalization by focusing more on representative samples.

To begin, detectors often consider hard negative samples as positive samples with high confidence. As a consequence, confidence is the most natural component to examine. The foreground intersection criteria^51^ is then used to assess an object’s incompleteness. Next, as shown below, we construct the scoring fusion function to account for the two aspects of IoF and confidence:

11 $$s_{i} = \frac{{e^{{\lambda C_{i} + (1 - \lambda )I_{i} }} }}{{\sum\nolimits_{i = 1}^{N} {e^{{\lambda C_{i} + (1 - \lambda )I_{i} }} } }}$$

where $C_{i}$ and $I_{i}$ represent the confidence of the $I_{i}$th detection result and the corresponding maximum IoF respectively, and λ represents the balance coefficient used to adjust the weight of IoF and confidence. We can then adjust the selection probability for each sample based on $s_{i}$.

#### Multi-object search non-maximum suppression(MO-NMS)

Non-maximum suppression (NMS) is a crucial post-processing step in object detection, streamlining the final results by filtering redundant detections. YOLOv8, like its predecessors, employs NMS for target localization. However, in complex scenarios like automated pear picking, where multiple pears cluster on a tree against diverse backgrounds, traditional NMS falls short.

To address this, this research proposes MO-NMS (Multi-Object Non-Maximum Suppression) for image post-processing in pear picking automation. Unlike standard NMS, Soft-NMS, or Matrix NMS, which operate locally on individual anchor boxes, MO-NMS takes a global perspective, filtering based on the relationships between all anchor boxes within the image. This approach aims to maximize both detection accuracy and minimize false positives, crucial for reliable pear picking. Instead of doing anchor box filtering from a local viewpoint like standard NMS, Soft-NMS, Matrix NMS, and other approaches, MO-NMS views it from a global perspective and conducts global screening based on the combination of anchor boxes. MO-NMS strives for the combined outcomes of high accuracy and low false detection in order to make it more appropriate for autonomous pear picking job situations. During the process of object detection, YOLOv8 generates anchor boxes of multiple scales and aspect ratios. Each box has a classification score and a regression offset, determined by softmax to determine whether the anchor box belongs to the foreground or the background. Then, the anchor boxes are adjusted using bounding box regression to obtain accurate proposals. All proposals are sorted in order of their scores, and the top K (≈6000) anchors are selected. YOLOv8 then employs standard Non-Maximum Suppression (NMS) techniques to find proposals with higher confidence among the anchors.

12 $$s_{i} = \left\{ {\begin{array}{*{20}l} {s_{i} ,} \hfill & {{\text{IoU}} \left( {M,a_{i} } \right) < N_{t} } \hfill \\ {0,} \hfill & {{\text{IoU}} \left( {M,a_{i} } \right) \ge N_{t} } \hfill \\ \end{array} } \right.$$

where $s_{i}$ is a re-scoring function, ${\text{IoU}}$ represents the inter-section area divided by the union area for two bounding anchor boxes, $a_{i}$ denotes the anchor box to be checked, $M$ is the anchor box with the highest score, and $N_{t}$ is a rigid threshold that determines whether an anchor should be retained or eliminated from the vicinity of $M$. Suppose $a_{i}$ represents a foreground region containing a small target. If $a_{i}$ has a significant overlap with $M$ beyond a threshold value $N_{t}$, the score $s_{i}$ of $a_{i}$ will decay to 0, indicating that the small target will be missed. Conversely, if $N_{t}$ is set too low, it may lead to the generation of false positives. In an attempt to enhance the Non-Maximum Suppression (NMS) process, Bodla et al. proposed the Soft-NMS algorithm^52^, which was applied to the region refinement stage. While this algorithm exhibits improved performance on natural images, experimental results have shown limited effectiveness in detecting small objects in optical remote sensing images.

Inspired by Soft-NMS, we propose a refined Non-Maximum Suppression (NMS) algorithm specifically designed for small targets, which we refer to as MO-NMS, named after its similarity to the sigmoid function in its key component. MO-NMS is applied in the region proposal stage and can be described as follows:

13 $$s_{i} = \left\{ {\begin{array}{*{20}l} {s_{i} ,} \hfill & {{\text{IoU}} \left( {M,a_{i} } \right) < N_{t} } \hfill \\ {\frac{{s_{i} }}{{1 + e^{{\eta {\text{IoU}} \left( {M,a_{i} } \right)}} }}} \hfill & {{\text{IoU}} \left( {M,a_{i} } \right) \ge N_{t} } \hfill \\ \end{array} } \right.$$

where η represents the attenuation parameter. The re-scoring function decays the scores of anchor boxes above a threshold $N_{t}$ using a penalty function based on their overlap with $M$. Anchor boxes that are distant from $M$ and have scores below $N_{t}$ remain unaffected. This update rule is applied iteratively, updating the scores of anchor boxes that meet the specified condition.

In conclusion, the proposed MO-NMS algorithm, which combines the characteristics of pear features with low background distinctiveness and small pixel occupancy, enhances the performance of the model in pear detection.

MO-NMS and Soft-NMS are two post-processing techniques used in object detection to filter out redundant bounding boxes. While both aim to improve detection accuracy, their approaches differ significantly. MO-NMS takes a global perspective, considering all anchor boxes simultaneously and employing a sigmoid-like function for rescoring, which gradually attenuates scores based on overlap. This makes it particularly effective in handling small objects and complex scenes where objects cluster together. In contrast, Soft-NMS focuses on local interactions between anchor boxes, applying a linear decay to scores. This approach might be less effective in handling small objects or crowded scenes.

Specifically in pear detection, MO-NMS shines due to its consideration of pear-specific features, such as their low contrast with the background and small size. By incorporating this domain-specific knowledge into its global filtering strategy, MO-NMS outperforms other NMS methods, including Soft-NMS, in terms of accuracy and reducing false positives. This enhanced performance translates to a more robust and reliable pear picking automation system.

## Results

This section will conduct experiments to validate the effect of HDMNet for pear object detection and compare it to other relevant models on the same test set. This part contains the following sections: experimental environment, experimental settings, HDMNet performance analysis, method effect analysis, comparison between SOTA models, ablation experiments, and practical application testing.

### Experiment environment

The tests in this research are done under the same hardware and software conditions to validate the performance of HDMNet described in this paper. See Table 1 for specific environmental parameters.

**Table 1:** Hardware and software environment.

Hardware environment	CPU	15 vCPU AMD EPYC 7543 32-Core Processor
	RAM	80GB
	Video memory	24 GB
	GPU	NVIDIA GeForce RTX 3090
Software environment	OS	Linux
	CUDNN V8.0.4;	
	CUDA Toolkit V11.6;	
	Python 3.8.1;	
	torch 1.8.1;torchvision 0.9.1	

### Experiment setting

The HDMNet model was trained using the Pytorch framework which loaded the pre-trained weights of YOLOv8 and the trained model weights were used to forecast the pear picture in order to validate the model’s efficacy and accuracy. An NVIDIA GeForce GTX 3090 graphics card and the Linux operating system were used to conduct the experiment.

We created and made public a dataset of automated pear picking prior to the commencement of this project. We randomly partition the data set into a training set, a verification set, and a set for testing to make sure that the properties of the pear itself and its flaws are fully learned. 7:1:2 for a test set. During training, a Gaussian distribution is used to initialize each layer of the model. We set the batch size to 16, the Momentum to 0.9, and the initial learning rate to 0.02 (the initial learning rate is proportional to the batch size setting) and adjusted it according to the SGD optimizer. The learning rate, Decay, and number of iterations are both set to 300 epochs. 20% of the self-made pear automated picking photos in this work are utilized as the test set during model testing and assessment. The experimental settings are shown in Table 2.

**Table 2:** Environment setting.

Size of input images	640 × 640	Initial learning rate	0.02
Batch_size	16	Decay	0.001
Momentum	0.9	Iterations	300 epochs

### Performance analysis of HDMNet

On the test set, we conducted a number of performance evaluation tests to show how HDMNet’s impact on YOLOv8 has improved. Because the threshold setting affects the experimental findings by changing the MO-NMS results, we display mAP, mAP50, mAP75, FPS, Params, GFLOPs and Infertime, HDMNet, and YOLOv8 in the test set to completely analyze our network model. Table 3 displays the comparative experimental findings on the aforementioned topics.

**Table 3:** Performance comparison of HDMNet and YOLOv8-s.

Evaluation	YOLOv8-s	HBAnet
mAP50	87.1	93.6
mAP75	63.5	70.2
mAP	68.2	75.7
mAP50(pear)	98.3	99.3
mAP50(stalk)	82.4	90.1
mAP50(claxy)	81.7	88.9
AR	45.8	48.6
F1	0.83	0.89
FPS	80.0	73.0
Params(M)	11.2	12.9
GFLOPs(G)	28.6	41.1

**Figure 4:**
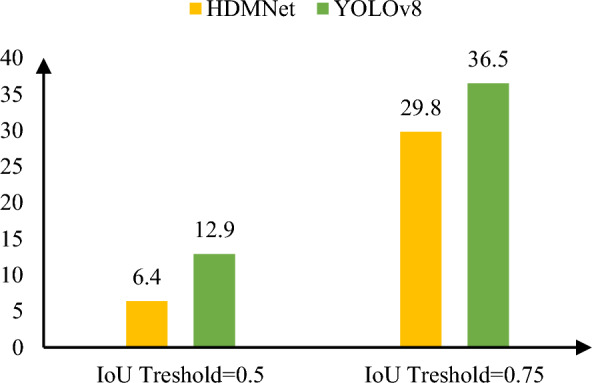
Comparison of false alarm rates under different cross-merge ratio settings.

This paper compares the false positive rates of HDMNet and YOLOv8-s under different IOU thresholds (see Fig. 4). Regardless of the threshold choice, HDMNet outperforms YOLOv8-s in terms of accuracy. HDMNet has improved feature extraction and feature fusion capabilities, however this is due to MO-NMS improvements. The anchor frame processing mechanism has been enhanced, resulting in greater positioning accuracy.

### Ablation experiments

We performed an HDMNet ablation experiment using A, B, and C to represent HAS, DP-FPN, and MO- NMS in order to test the efficacy of the method suggested in this paper. The experimental results are displayed in Table 4 for each improvement that was verified using the YOLOv8-s framework.

**Table 4:** Ablation experiment results.

Group	Method	mAP	mAP^50^	mAP^75^	AR	FPS	Param	GFLOPs
1	YOLOv8-s	68.2	87.1	63.5	45.8	80.0	11.2M	28.6
2	YOLOv8-s + A	71.0	89.9	66.2	46.9	76	12.2M	36.2
3	YOLOv8-s + B	70.1	89.2	65.4	46.6	76.1	11.9 M	33.4
4	YOLOv8-s + C	69.7	88.8	65.0	46.2	79.6	11.2M	28.8
5	YOLOv8-s + A + B	74.0	91.6	67.9	47.9	73.4	12.9 M	40.9
6	YOLOv8-s + A + C	71.7	89.5	68.1	47.7	75.7	12.2 M	36.4
7	YOLOv8-s + B + C	71.3	89.1	67.7	47.3	75.6	11.9M	33.7
8	HDMNet	75.7	93.6	70.2	48.6	73.0	12.9 M	41.1

Based on YOLOv8, we add HSA, DP-FPN, and MO-NMS using the control variable technique. By merging the improvements of these three modules with HDMNet, eight sets of ablation experiments were performed. Of them, HSA has the most significant improvement in mAP, RDDFPN has a substantial improvement on long-distance pear targets, and MO-NMS has a little contribution to mAP improvement, which improves positioning accuracy. The ablation tests demonstrate that the HDMNet enhancement has a considerable improvement in target identification accuracy in autonomous pear picking situations. In summary, HDMNet grew by 7.5% in mAP and 6.5% in mAP50 when compared to YOLOV8-s. In terms of speed and parameter volume, the YOLOv8-s pipeline is still a fast and lightweight network that fulfills real-time detection criteria due to its simplicity. The comparative results of 8 sets of tests can completely demonstrate the function of the three techniques described in this study in enhancing the model’s accuracy in autonomous pear picking. As a result, we feel that HDMNet is more suited than YOLOv8-s for target identification in autonomous pear picking settings.

### Visualization of detection results

The visual reasoning outputs of the object detection model must be studied in order to further intuitively assess HDMNet. We illustrate the enhanced identification results of YOLOv8-s based on the eight ablation tests listed in Table 4. The identified frames, categories, and confidence levels are shown on the detection result graph, as shown in Table 5.

**Table 5:**
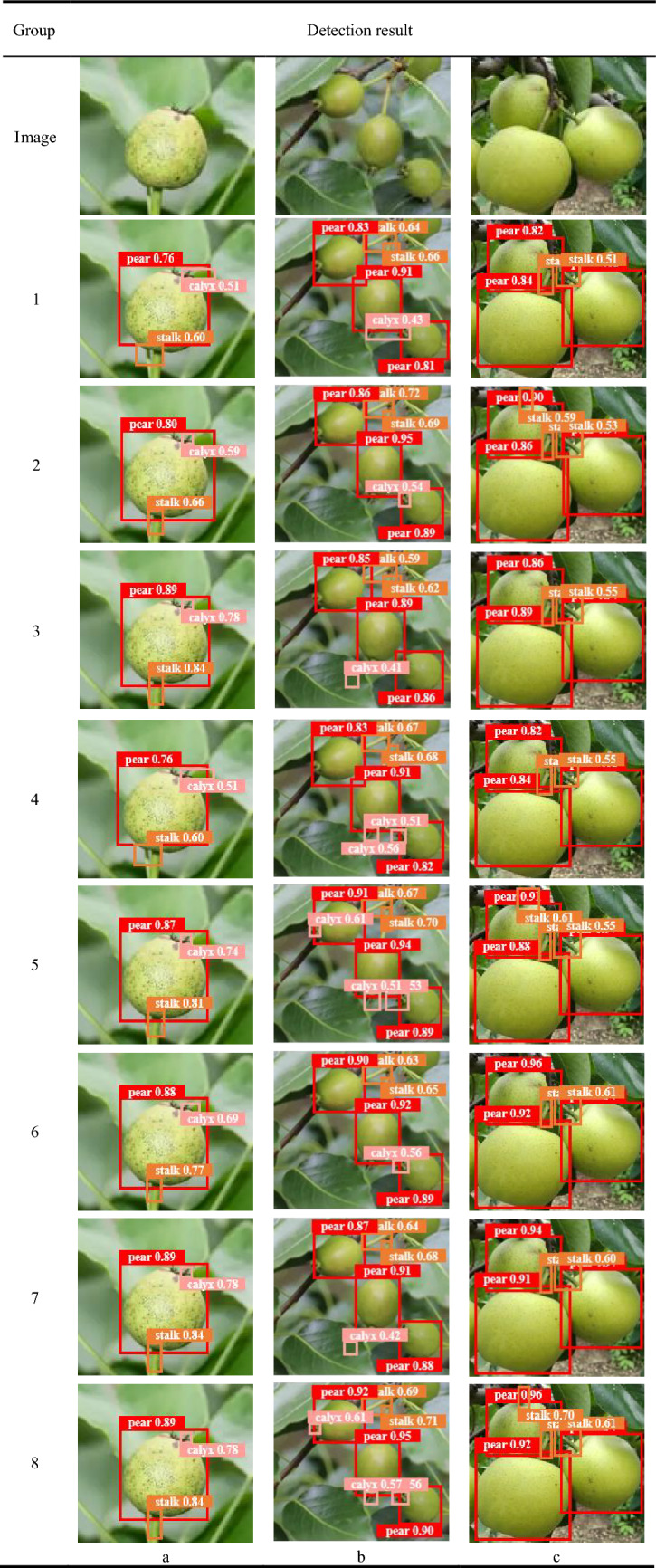
Visual comparison of test results.

From Table 5, we can see that for Figure a, the fruit pear has a color similar to that of the surrounding leaves, while the features of the calyx in the pixels are not obvious. Although YOLOv8-s detected the fruit stalk and fruit calyx, the positioning frame Very inaccurate, the surrounding background is detected, resulting in the area of the detection frame being significantly higher than the actual area of the target, and has a lower confidence. The detection frame is made to fit the pear component (Group 2) by HAS’s extraction of more precise pear features. In addition to improving the fusion of pear features, DP-FPN also achieves a better detection position (Group 3). MO-NMS accurately selected the combined results, but this was not evident in the detection results of individual pears (Group 4). For panel b, the detection of interlaced calyxes on the lower two pears is a challenge. HSA strengthens feature extraction and increases detection confidence (Group 2). DP-FPN is good at small object detection, but without the support of HAS, it is also easy to misidentify the background (Group 3). MO-NMS optimizes the results of the combination of anchor boxes, which can improve the detection effect of these two calyxes (Group 4). For Figure c, the stem feature in the upper left corner is not obvious and occupies very few pixels. When YOLOv8-s adds HAS and DP-FPN (Group 5), the position can be detected, but the positioning and confidence need to be improved. When MO-NMS is added on this basis, the positioning accuracy and confidence are both there is further improvement (Group 8).

**Figure 5:**
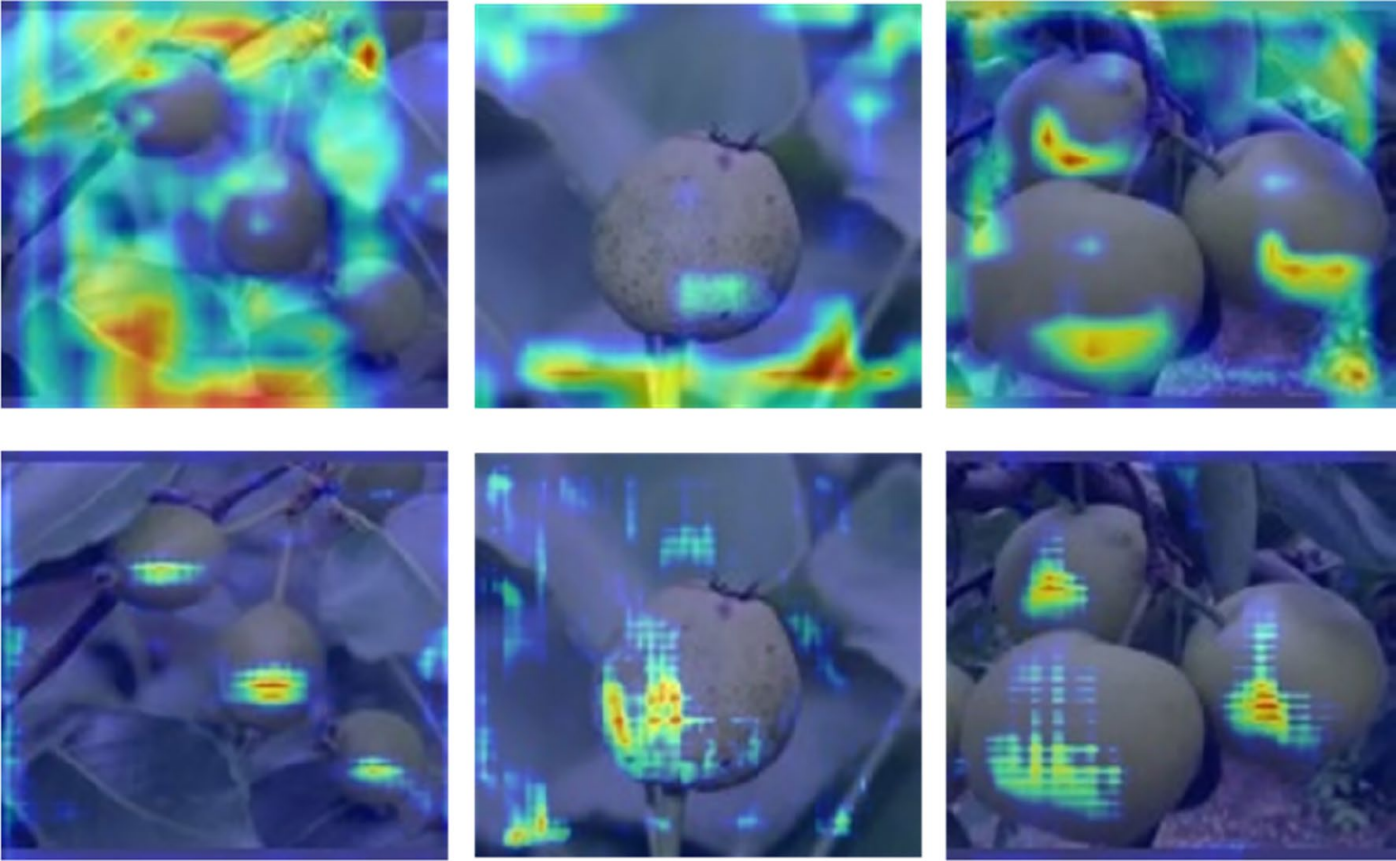
The heatmap of the backbone (the first line represents YOLOv8, the second line represents HDMNet).

In order to explore the key to the performance improvement of the model, we compared the feature map visualization information of the heatmap of HDMNet and YOLOv8. The results are shown in Fig. 5, which shows that HDMNet has rich feature information compared with YOLOv8.

From the results, we can see that our proposed HDMNet can focus on the pear object, thereby significantly improving the model performance.

### Method effect analysis

The effects of the designed HSA, DP-FPN, and MO-NMS are described in detail in this section, and their performance is examined when they are added to or subtracted from the corresponding positions of YOLOv8 (where HAS is added at the end of the backbone, DP-FPN directly replaces the FPN structure, and MO-NMS is used as the anchor box selection criterion for new image post-processing). In the experiment, in order to interfere with the experiment caused by the input image enhancement and the inconsistent calculation of the loss function, we adopted the control variable method and still used the image enhancement and loss function that comes with the YOLOv8 pipeline. The main results of the comparative experiment are as follows:

#### High-level Semantic focused attention mechanism module(HSA)

Due to complex background interference, the Backbone stage of YOLOv8 does not fully extract the features of pears. Here, this paper proposes the feature information of HSA enhanced extraction. In order to prove the effectiveness of HSA in enhancing features and the separate role of its multi-scale convolution and Semantic focusing components, we removed it in HDMNet to explore the gain brought by HSA to the model. The experimental results are shown in Table 6.

**Table 6:** Exploration of HSA ablation experiment.

Method	mAP	mAP^50^	mAP^75^	AR	FPS	Param	GFLOPs
HDMNet	75.7	93.6	70.2	48.6	73.0	12.9 M	41.1
Using single-scale convolution	74.8	92.5	68.7	48.2	73.6	12.7M	36.8
Remove emantic focus component	72.1	91.1	69.3	47.6	75.1	12.1M	37.2
Remove HSA	71.3	89.1	67.7	47.3	75.6	11.9M	33.7

It can be seen from the data in the table that when the first and second parts of the HAS are removed, all accuracy indicators of the model are affected. It can be seen from the data that the first part determines the multi-scale information of the model, which determines the model. Confidence has a greater impact on mAP75. The second part determines the key feature extraction of the model, which greatly affects the mAP and mAP50 indicators. It turns out that HSA plays an important role in feature enhancement in the feature extraction stage.

#### Deformation-perception feature pyramid network(DP-FPN)

DP-FPN consists of three parts: scale enhancement module, scale selection module, and weighted negative sampling. These three parts are referred to as A, B, and C in the table below. In order to explore the best combination of DP-FPN, remove the components one by one on the basis of HDMNet, and conduct ablation experiments to explore the effect of DP-FPN. The results are shown in Table 7.

**Table 7:** Exploration of ablation experiment of RDDFPN.

Method	mAP	mAP^50^	mAP^75^	AR	FPS	Param	GFLOPs
HDMNet	75.7	93.6	70.2	48.6	73.0	12.9 M	41.1
Remove A	74.0	92.7	70.0	48.4	73.6	12.5M	39.9
Remove B	74.2	92.9	69.8	48.3	73.4	12.5M	39.6
Remove C	74.1	92.5	69.9	48.3	73.1	12.9M	38.9
Remove A + B	72.3	91.1	68.8	48.1	74.8	12.2 M	37.2
Remove A + C	72.4	90.9	68.6	48.0	74.7	12.5 M	37.5
Remove B + C	72.2	90.8	68.9	47.9	74.9	12.5M	37.7
Remove A + B + C	71.7	89.5	68.1	47.7	75.7	12.2 M	36.4

It can be shown that improving feature fusion leads to an increase in detection accuracy. When all three components are employed, the model’s accuracy improves the highest when compared to the other seven combinations, particularly for the changing goal size of fruit pear detection.

#### MO-NMS

**Figure 6:**
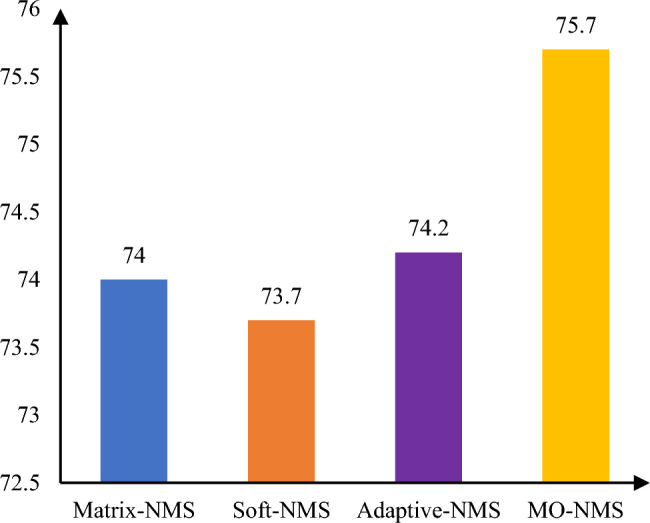
Comparison of the effects of different NMS methods.

This paper proposes MO-NMS for object detection in the automated picking of pears. In order to explore the effectiveness of MO-NMS, the NMS of the HDMNet model is replaced. Figure 6 depicts the performance of Matrix-NMS, Soft-NMS, Adaptive-NMS, and MO-NMS on the mAP index.

It can be seen that, compared with Matrix-NMS, Soft-NMS and Adaptive-NMS, MO-NMS is more conducive to improving the recognition and positioning accuracy in the object detection task in the automated pear picking scene.

### Comparison between SOTA models

This research compares a few traditional and contemporary sophisticated object identification methods with the proposed HDMNet on the same test environment and test set in order to validate the detection performance of the HDMNet model. Table 8 displays the mAP, mAP50, mAP75, AR, and FPS of various networks on the test set.

**Table 8:** HDMNet outperforms other detectors in the self-made data set test of automated pear picking in this paper.

Method	Backbone	mAP	mAP50	mAP75	AR	FPS
Two-stage detectors						
Faster R-CNN	Resnet50	59.6	81.8	58.1	40.6	15.1
Faster R-CNN	ResNet101	61.1	83.3	60.3	41.5	10.6
Mask R-CNN	ResNet101	61.6	83.8	60.9	41.9	8.9
Cascade R-CNN	ResNet101	63.7	84.2	60.2	43.0	4.8
Libra R-CNN	RseNext101	66.1	86.0	61.3	45.4	5.5
SINPER	ResNet101	66.6	86.4	62.0	45.7	5.3
Cascade Mask R-CNN	ResNet152	69.7	88.2	64.5	46.6	3.9
One-stage detectors						
SSD512	VGG16	58.7	80.0	57.6	40.2	91.8
RetinaNet	ResNeXt101	66.1	85.0	62.8	44.2	66.2
RefineDet	ResNet101	65.8	85.4	62.1	44.8	62.6
CornerNet	Hourglass104	64.6	84.7	60.6	44.0	24.9
FSAF	ResNext101	62.0	82.2	58.9	42.1	68.5
YOLOv3-s + ASFF	Darknet53	63.6	83.7	59.4	43.1	68.2
YOLOv4-s	CSPDarknet53	64.8	84.6	60.1	44.2	77.9
YOLOv5-s	Modified-CSP v5	64.9	84.8	60.2	44.1	111.2
YOLOv6-s	Modified-CSP v6	66.5	85.6	61.1	45.0	82.2
YOLOv7-s	Modified-CSP v7	67.9	86.8	63.0	45.6	78.8
YOLOv8-s	Modified-CSP v8	68.2	87.1	63.5	45.8	80.0
Transformer-based detectors						
Deformable DETR	Swin-Transformer	70.1	89.2	65.6	47.1	**–**
Cascade R-CNN	Swin-Transformer	69.2	89.0	64.5	46.7	**–**
Deformable DETR	PVTv2	71.2	90.0	66.1	47.3	
Cascade R-CNN	PVTv2	70.0	89.1	64.2	46.9	**–**
Ours						
HDMNet	Modified-CSP v8 + HSA	75.7	93.6	70.2	48.6	73.0

HDMNet outperforms all other detectors in detection accuracy and achieves competitive results with numerous single-stage detectors in real-time.

As is commonly acknowledged, the single-stage detector has better real-time performance and lower parameter quantity than the two-stage detector, which is suitable for the timeliness and deployment cost of automated pear picking. For the above two-stage detector, although it shows relatively good accuracy, it is not effective in real-time performance, and the improvement in accuracy is not obvious, so it is not used as the baseline of this article. Although SSD512, RetinaNet, RefineDet, CornerNet, and FSAF for single-stage detectors offer faster detection speeds than two-stage networks, they are less accurate. The YOLOv3 ~ YOLOv8 model has gradually improved its performance with the development, which has been verified on the data set of this article, which is why the baseline of this article chooses YOLOv8-s. For Transformer-based detectors, the amount of parameters and calculations are large, and the real-time performance is not even as good as the two-stage model, which is not suitable for the object detection of automated pear picking tasks. For the improvement of our proposed HDMNet based on YOLOv8-s, from the results, HDMNet is superior to all models used for comparison in mAP, mAP50, and mAP75, and it is second only to a few models in FPS. Compared with YOLOv8-s, the gap is not obvious, and it can still meet the real-time requirements of automated pear picking tasks. In summary, HDMNet is the most suitable model for object detection in the automated picking of pears. We analyzed why our proposed HDMNet is superior to other deep neural network models, and the reasons are as follows: (1) HDMNet is improved based on YOLOv8-s. YOLOv8-s itself uses a concise pipeline, which can ensure strong real-time performance and low parameters. (2) The proposed HAS is added to the end of the backbone of HDMNet, which optimizes the response of fruit and pear features that are not obvious in the feature map, and greatly improves the extraction effect of fruit and pear features in complex scenes. (3) DP-FPN improves the feature fusion ability of multi-scale fusion, which not only enables the model to adapt to the fusion of different scale features, but also is more suitable for the positioning of pears in complex scenes. (4) MO-NMS improves the anchor box selection strategy, and obtains a set of screening anchor boxes through the search method based on the objective function. When there are multiple pear targets, better positioning can be obtained.

### Practical application test

On the one hand, the Internet of Things system is susceptible to interference caused by poor signals in the orchard, which affects the transmission of pictures and the final detection effect of the model; on the other hand, a good pear detection model should not be tested only in ideal orchard environments with excellent lighting conditions but also needs to be evaluated in more typical and complex orchard settings. This is because practical application environments may involve more common environmental disturbances such as shadows, varying lighting, and obstructions. To validate the model’s generalization capability, we organized the capture of 300 real-life pear orchard images in Yikang Fruit Farm, Chudong Village, Daweishan Town, Liuyang City, Hunan Province. Additionally, we established an Internet of Things (IoT) system utilizing a camera as the image input device.

**Figure 7:**
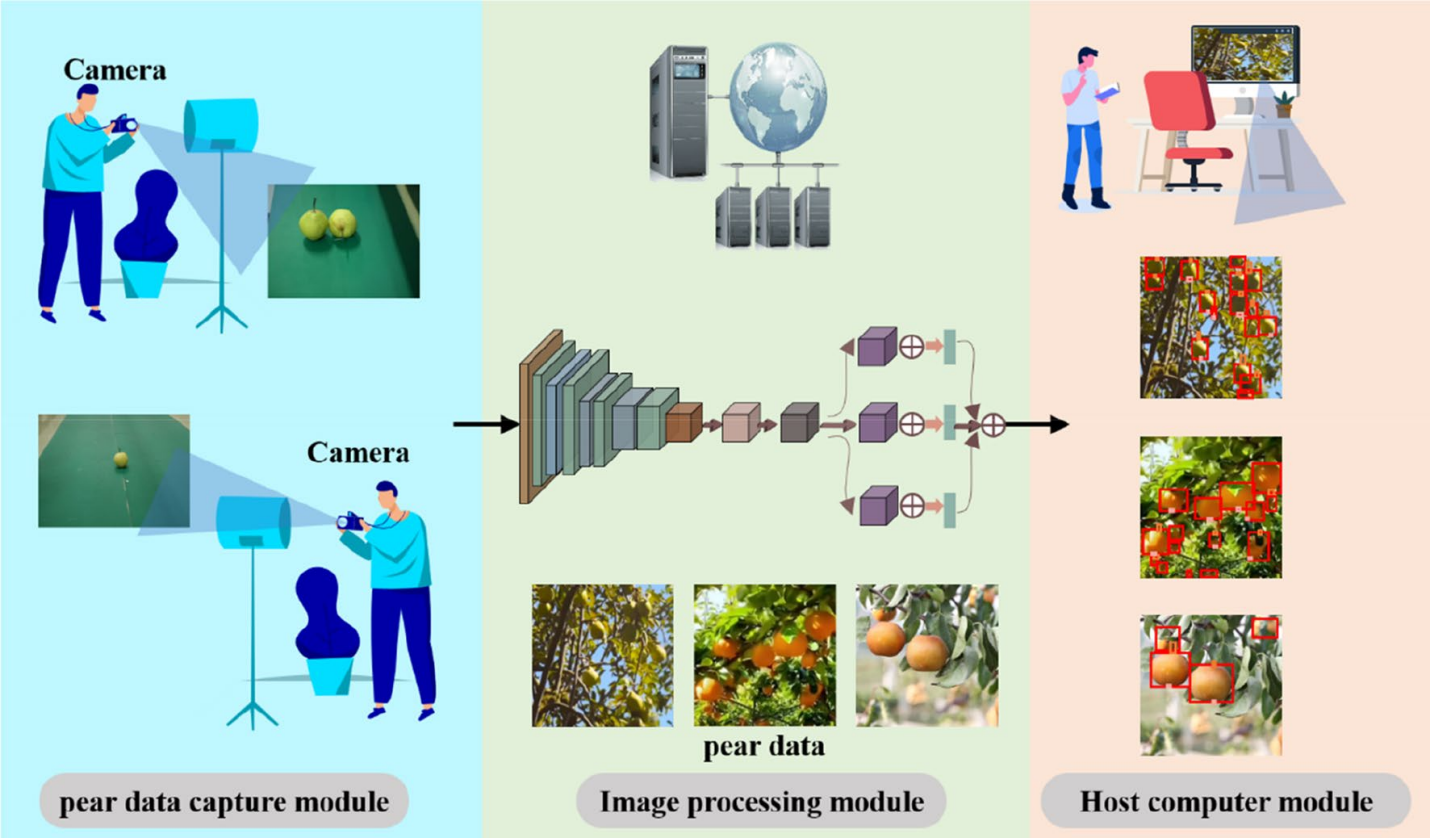
Schematic diagram of the automated picking detection of pear detection system of the IoT based on HDMNet.

Considering that the shooting equipment in real application scenarios may not be uniform, we used multiple cameras of different models for data collection to reduce the impact of differences in data collection equipment on the results. The process is shown in Fig. 7.

To tackle signal and environmental issues in pear orchards, the IoT system uses highly sensitive cameras adaptable to changing light. These cameras adjust to shadows and sunlight, vital in outdoor farms. The mesh network design improves data reliability, letting devices connect with many nodes. This allows data rerouting if nodes fail or signals are blocked, keeping the network working throughout the orchard. Edge computing is used to process data near the cameras, lowering data sent to main servers and speeding up processing. This includes initial image analysis, important for data and quick decisions. The system is secure, using encrypted communication and safe storage to prevent data leaks. It’s also flexible, allowing new technology and sensors, ensuring it can grow and change with farming needs. This not only makes pear detection and health checks better but also strengthens the system against common problems in farm IoT use.

**Figure 8:**
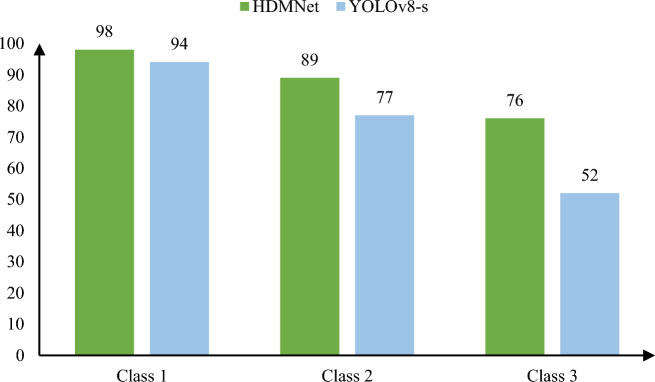
Detection results of HDMNet and YOLOv8-s in three types of pictures: Category 1 refers to close-range targets with obvious features, category 2 refers to mid-range targets, and features that are not significant; category 3 refers to long-distance and difficult-to-recognize features.

Figure 8 depicts a comparison of YOLOV8-s and HDMNet recognition in three types of pear detecting settings. We grouped 300 genuine scenes shot in Yikang Fruit Farm, Chudong Village, Daweishan Town, Liuyang City, Hunan Province, into three categories for testing. The chart shows that YOLOV8-s and HDMNet have 94% and 98% identification accuracy rates in category 1, respectively, 89% and 77% recognition accuracy rates in category 2, and 76% and 52% recognition accuracy rates in category 3.

To demonstrate the benefits of HDMNet in actual application settings, we chose the detection results of an image from each of the three conditions to present. In these scenarios with a dense and numerous set of targets, we have omitted categories and confidence scores to ensure the readability of the images, preventing excessive labels from obscuring the objects themselves. The detection outcomes for the two models across three scenarios are shown in Table 9.

**Table 9:**
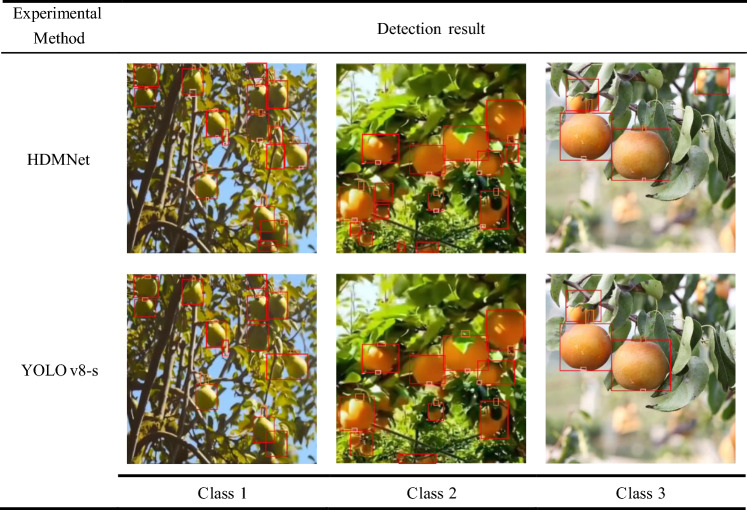
Detection results of the two models

It can be seen that for category 1, HDMNet’s detection frame is more in line with the actual position of the pear than YOLOv8-s, while the fruit stalk with inconspicuous features is also detected. For category 2, HDMNet eliminates false detections brought by capturing shadows. For category 3, HDMNet detected long-distance stalk targets, while YOLOv8-s failed to detect them. In summary, HDMNet has high precision and positioning accuracy for these three situations. The results of the real operations point to the ability of the methodology proposed in this study to accurately locate the position of the pear and its essential parts, which is crucial for autonomous pear picking and agricultural automation.

## Discussion

We have produced an object detection data set with a total of 8363 images for object detection of automated pear picking, which covers assorted characteristics of various varieties of mature pears captured by cameras at different distances during pear picking. Through the comparison and analysis of various sets of trials, the effectiveness of the suggested HDMNet for automated pear picking is confirmed. It addresses the three key issues of complicated backdrop interference and leaf occlusion, low detection accuracy of long-distance pears, and erroneous placement of many pears in particular.

Image or video databases of common things are currently regularly utilized. Unfortunately, there is a gap in agricultural automation, therefore we created our own dataset of automated pear picking photos collected by cameras and annotated with labelIing. We intend to give some help to other researchers in the field of agricultural automation through the dataset and share it to the training and testing of the HDMNet model.

We have established an Internet of Things system for automated picking and detection of pears, which involves the joint application of cameras and servers. In practical applications, the picking robot can jointly build a corresponding multi-point network system, which can detect the position of the fruit pear on the fruit tree and the position of the fruit stem and fruit calyx relative to the fruit pear in real time, and can respond and process effectively. When the detection is completed, according to the location of the detection model on the server, the position of the anchor frame will be further fed back to the picking robot, so as to achieve a stable and precise robotic arm grabbing process.

## Conclusion & outlook

In recent years, with the increase in agricultural output and the loss of agricultural population, agricultural labor has become in short supply, consequently the development of deep learning technology has given new inspiration to agricultural automation technology^53^. We proposed HDMNet for auxiliary object detection for automated pear picking, to achieve high-precision and accurate positioning targets, reduce the loss of automated pear picking, and realize agricultural automation and modernization to the greatest extent. First of all, we propose HSA, which combines the feature that the pixel-based features of pear are not obvious, enhances the activation value of the feature map, and effectively obtains the feature Semantic information of the model. Then, in order to further release the gain brought by the attention mechanism module, we propose DP-FPN, which can effectively fuse feature information of different scales. Finally, in the scene of automated picking of pears, the environment of the pears is uncertain, and the pears on the fruit tree often have multiple characteristics. Search with a given objective function to achieve a combined result with high precision and low false detection. More importantly, HDMNet improves the accuracy and effectiveness of the YOLOv8 model in automated pear picking detection, and is easy to train and use. This opens new avenues for using deep learning in agricultural automation tasks. In our experiments, we use the ratio of 7:1:2 to divide the 8363 image data assembled by ourselves into a training set, validation set and test set. HDMNet achieves mAP50 of 93.6% and FPS of 73, thus outperforming other SOTA methods and demonstrating the effectiveness of the model we developed.

The experimental results show that the proposed HDMNet object detection method can effectively detect the pears on the fruit tree, but if it is put into practical application, the model in this paper still needs an investment to build the model on the server, and its large-scale application in the agricultural automation industry There is still a gap, the main reason is that the model proposed in this paper has a lower amount of parameters and calculations than most object detection methods. However, in the agricultural automated production line, in order to ensure low cost and high popularity, the parameters of the model should be compressed to less than 4 M, and have a good CPU calculation speed. However, such model design conditions are harsh and may affect detection accuracy, and there are also a lot of other considerations.

In the future, we will further compress the model, explore the balance between model deployment simplicity and detection accuracy, and pursue a low-cost usage method to facilitate large-scale deployment in real agricultural automation production applications.

## Data Availability

The data presented in this study are available on request from the corresponding author. The data are not publicly available due to partial authors’ disagreement. The datasets used and/or analyzed in this study have all been uploaded to the website.
